# Resource use and associated energy expenditure of African crowned eagles (*Stephanoaetus coronatus*) in an urban-forest mosaic landscape

**DOI:** 10.1093/conphys/coag068

**Published:** 2026-09-18

**Authors:** Lara Howard, Varalika Jain, Shane C Sumasgutner, Jesús Bautista, Ralph Buij, Colleen T Downs, Susan J Cunningham, Petra Sumasgutner

**Affiliations:** FitzPatrick Institute of African Ornithology, Department of Biological Sciences, University of Cape Town, Private Bag X3, Rondebosch, Cape Town 7701, South Africa; Konrad Lorenz Research Center for Behavior and Cognition, Core Facility of the University of Vienna, Fischerau 13, Grünau im Almtal 4645, Austria; Konrad Lorenz Research Center for Behavior and Cognition, Core Facility of the University of Vienna, Fischerau 13, Grünau im Almtal 4645, Austria; Department of Behavioral and Cognitive Biology, University of Vienna, Djerassiplatz 1, Vienna 1030, Austria; Konrad Lorenz Research Center for Behavior and Cognition, Core Facility of the University of Vienna, Fischerau 13, Grünau im Almtal 4645, Austria; Centre for Functional Biodiversity, School of Life Sciences, University of KwaZulu-Natal, Carbis Road, Scottsville, Pietermaritzburg 3209, South Africa; Wilder South NGO, Camino de San Antonio No. 9, Cármenes de Aynadamar, House 16, Granada 18011, Spain; Animal Ecology Group (AEG), Wageningen Environmental Research, PO Box 47, Wageningen 6700 AA, The Netherlands; The Peregrine Fund, 5668 West Flying Hawk Lane, 83709 Boise, ID, USA; Centre for Functional Biodiversity, School of Life Sciences, University of KwaZulu-Natal, Carbis Road, Scottsville, Pietermaritzburg 3209, South Africa; FitzPatrick Institute of African Ornithology, Department of Biological Sciences, University of Cape Town, Private Bag X3, Rondebosch, Cape Town 7701, South Africa; FitzPatrick Institute of African Ornithology, Department of Biological Sciences, University of Cape Town, Private Bag X3, Rondebosch, Cape Town 7701, South Africa; Konrad Lorenz Research Center for Behavior and Cognition, Core Facility of the University of Vienna, Fischerau 13, Grünau im Almtal 4645, Austria; Department of Behavioral and Cognitive Biology, University of Vienna, Djerassiplatz 1, Vienna 1030, Austria

**Keywords:** Animal movement, apex predator, bioenergetics, biologging, overall dynamic body acceleration (ODBA), physiological costs, urban exploiter

## Abstract

Rapid urbanization is a major driver of global change, making it increasingly important to understand how wildlife persists in highly transformed landscapes. Using insights from movement (Global Positioning System, GPS) and activity (accelerometry, ACC) data, we aimed to understand the behavioral and physiological mechanisms associated with an apex predator's successful exploitation of heavily transformed landscapes. The African crowned eagle (Stephanoaetus coronatus), a subtropical forest eagle, breeds at unusually high densities in Durban, eThekwini Municipality, South Africa. Here, crowned eagles inhabit an urban–forest mosaic, much of which falls within the Durban Metropolitan Open Space System (D'MOSS), a network of protected and semi-natural green spaces embedded within the city. Using data from five GPS/ACC-tagged crowned eagles, we assessed individual resource selection and determined how energy expenditure, inferred from overall dynamic body acceleration, varied across habitat types and with distance from the D'MOSS boundary. We found that crowned eagles selected forested habitats over built-up habitats when available. Energy expenditure was higher in built-up environments and increased with distance from the D'MOSS boundary. Our findings suggest that habitat structure may influence energy costs, although quantifying these relationships in heterogeneous urban landscapes remains challenging. We underscore the importance of protected habitat networks in cities and provide insights into the physiological costs of navigating urban–forest mosaic landscapes. These insights can inform urban planning to conserve natural habitat networks and ecosystem features that support the persistence of urban biodiversity, including apex predators.

## Abbreviations

ACC, Accelerometry
AKDE, Autocorrelated kernel density estimate
D’MOSS, Durban Metropolitan Open Space System
GPSn, Global Positioning System
ODBA, Overall dynamic body acceleration.

## Introduction

Urbanization is a major driver of global change, transforming ecosystems at an unprecedented scale and pace (Grimm *et al.*, 2008). Urban development often results in habitat fragmentation, which alters the quality, shape and size of habitat patches (LaPoint *et al.*, 2015), thereby redistributing resources across the landscape (Lowry *et al.*, 2013) and influencing how species move between patches (Teitelbaum *et al.*, 2020). Consequently, urban environments may function as varied ‘energetic landscapes’, in which spatial variation in habitat structure, resource availability and anthropogenic disturbance alters the energetic costs of animal movement and foraging (Shepard *et al.*, 2013). Understanding how different habitats across urban landscapes shape an animal’s allocation of energetic resources is therefore essential for identifying habitat features that mitigate physiological stress and support viable populations in cities. In this sense, conservation physiology can identify the mechanistic links between environmental change and individual performance thereby informing management and planning (Wikelski and Cooke, 2006). In urban systems, this requires integrating physiology, behavioural ecology and spatial planning to assess not only where animals occur but also the energetic consequences of their habitat use.

Movement, a key behavioural mechanism through which animals respond to environmental change and acquire resources, is fuelled by energy (Butler *et al.*, 2004; Abrahms *et al.*, 2021). Variation in the energy expenditure associated with movement patterns can be used to estimate the physiological demands experienced by individuals in navigating different environments. For instance, elevated energy expenditure can reduce body condition, compromise reproductive investment and ultimately affect population persistence (Wilson *et al.,* 2008; Halsey *et al.*, 2011; Cooke *et al.*, 2013). This is particularly relevant in urban contexts, where species encounter anthropogenic barriers to movement and dispersal, such as roads (Bennett, 2003; Poessel *et al.*, 2014), potentially increasing movement complexity and associated energetic costs.

Among the species most affected by urbanization are apex predators, which have extensive resource requirements and depend on large, connected areas to establish territories and forage for sufficient prey (Fischer *et al.*, 2012; Sumasgutner *et al.*, 2014; Streicher *et al.*, 2021). As a result, apex predators are expected to experience pronounced physiological and behavioural challenges in fragmented urban landscapes. Furthermore, since apex predators exert strong top-down effects on ecosystems (Paine, 1980), changes in their behaviour or physiology may influence broader ecosystem processes. Understanding the physiological constraints of urban predators is therefore particularly important for designing protected habitat networks within cities that can sustain predator populations and their ecological functions (Reynolds *et al.*, 2021).

Recent developments in biologging have advanced the capacity to collect and integrate information regarding an animal’s movement, physiology and external environment. Remote sensing devices, such as Global Positioning System (GPS)-trackers, are increasingly accessible and can obtain high-resolution, real-time data of multiple different forms from a single device. One important advancement is the incorporation of tri-axial accelerometers, which record acceleration across three spatial axes. Tri-axial accelerometry (ACC) data can be used to estimate overall body dynamic acceleration (ODBA). ODBA is a widely used and extensively validated proxy for energy expenditure across vertebrate taxa (Wilson *et al.*, 2008, 2020; Halsey *et al.*, 2009). By integrating behavioural information from active movement with physiological proxies of energy expenditure, biologging allows an increasingly comprehensive assessment of how animals interact with human-modified environments.

A unique study system is provided by the African crowned eagle (*Stephanoaetus coronatus*), a large-bodied forest raptor that breeds at high densities throughout the urban-forest mosaic landscape of Durban, Ethekwini Municipality, South Africa (McPherson *et al.*, 2016a; Maseko *et al.*, 2023). Most urban raptors are small-bodied and habitat generalists (Cooper *et al.*, 2022; Headland *et al.*, 2023) and so the crowned eagle presents a valuable study system to investigate the success and persistence of a large and powerful, forest-specialist apex predator in an urban landscape mosaic. The species’ success in Durban has been largely attributed to the conservation of natural forest patches through the Durban Metropolitan Open Space System (D’MOSS; Roberts, 1994; Downs *et al.*, 2021), a municipal spatial planning framework designed to support biodiversity, habitats and ecosystem functioning (Reynolds *et al.*, 2021). Crowned eagles breeding within Durban must therefore navigate a complex mosaic landscape of hazards and resources (McPherson *et al.*, 2021). Previous research has highlighted behavioural plasticity in this population, e.g. the shift from biennial to annual breeding and urban-associated changes in the diet of this ambush predator (McPherson *et al.*, 2016a, 2016b; Muller *et al.,* 2020), and suggested a high tolerance to human activity (McPherson *et al.*, 2016a). However, little is known about the physiological costs and trade-offs associated with its successful exploitation of a major metropolis.

We used high-resolution GPS-tracking and ACC data from five crowned eagles to determine the extent of resource use and the energy expenditure patterns associated with different habitats. Inferring from earlier results of research on home-range and habitat selection (McPherson *et al.*, 2016a; Maseko *et al.*, 2023), we expected that crowned eagles would preferentially select for forest habitats rather than available urban built areas within the mosaic urban landscape. We hypothesized that the energy demands of different habitats within the landscape will vary, with urban habitats imposing higher relative energetic costs when compared with natural forest habitats, due to increased exposure to hazards. Anthropogenic hazards are expected to elevate energy expenditure in urban raptors through three, non-mutually-exclusive mechanisms: (i) higher rates of alert and flight-initiation responses to humans, vehicles and domestic animals (Frid and Dill, 2002; Møller, 2008); (ii) increased reliance on energetically costly powered flight when crossing built-up matrix where thermal updrafts are weak, unpredictable or turbulent (Shepard *et al.*, 2013); and (iii) chronic shifts in time and activity budgets consistent with a physiological landscape of fear (Gaynor *et al.*, 2019). Here, we expect flight-related responses to result in higher energy expenditure and increased ODBA values. In large-winged soaring birds, simultaneous heart-rate calibrations have confirmed that ODBA captures energetic variation during flapping flight, however underestimates costs during sustained soaring (Spivey and Bishop, 2013; Duriez *et al.*, 2014). Importantly, as forest-specialized raptors with relatively short wings and large bodies, the crowned eagle is expected to rely predominantly on perch-based hunting and short flapping flights through forested habitat, with flight movement comprising only a minor component of its daily activity budget (cf. forest-dwelling accipitrids spend the vast majority (>90%) of daylight perched, with only ~6–10% devoted to active flight, and soaring representing yet another fraction of this flight time; Widen, 1984; Rutz, 2006), consistent with preliminary observations from this population (Supplementary Tables S1 and S2). We therefore expected increased ODBA to appropriately capture energetically expensive flight responses in crowned eagles. To explore the role of anthropogenic disturbance, we used two indicators of development: the distance to the nearest road, and the distance to the D’MOSS boundary. Inside the D’MOSS, we predicted energy expenditure would decrease with increasing distance to the boundary, as crowned eagles move further away from higher densities of human disturbance. Outside of the D’MOSS, we predicted an increase with increasing distance to the boundary, due to growing anthropogenic disturbance.

## Materials and methods

### Study site and species

The city of Durban on the southeastern coast of the province of KwaZulu-Natal is South Africa’s third largest city with roughly four million residents (eThekwini Municipality, 2023). The climate is humid and subtropical, experiencing an annual average rainfall of 1015 mm, most of which (~80%) occurs within the summer months (Engelbrecht *et al.*, 2025). Durban’s varied topography includes three of South Africa’s nine biomes—Forest, Savannah and the Indian Ocean Coastal Belt. As a result, Durban is home to an exceptional diversity of species, many of which are heavily reliant on the habitat patches connected by the D’MOSS (eThekwini Municipality, 2023). The D’MOSS encompasses roughly 950 km^2^ of ecologically viable green space (eThekwini Municipality, 2023), of which only 7.25% is under formal protection. This lack of protection means it is highly heterogeneous and interspersed with urban development such as residential areas, recreational infrastructure and roads, and is subject to anthropogenic pressures such as residential development and unsustainable natural resource harvesting (eThekwini Municipality, 2023). Despite these pressures, the majority of crowned eagle nests in this study (seven of eight nest locations from five breeding adults; Supplementary Fig. S1), and 95% of those occurring within the greater Durban metropolitan area (McPherson *et al.*, 2016a) are located within the D’MOSS boundaries.

### Data collection

Between October 2023 and January 2024, five breeding adults (two females and three males) from five unique territories, were captured using a carcass-baited leg noose trap close to their respective nests (McPherson *et al.*, 2019). Trapping attempts were made when the chicks of the respective breeding adults were between 35 and 60 days old, under suitable weather conditions (i.e. avoiding extreme heat and heavy rain). Eagles were equipped with Ornitela 50 g solar powered GPS tags that transmit data via the Global System for Mobile Communications. Transmitters were fitted using a consistent backpack harnesses design, with Teflon ribbon straps connecting from the device at the back to a deteriorating ‘weak link’ loop (made of leather) at the front, which lasts ~5–7 years. All transmitters had neoprene padding at the base and straps were secured with aluminium crimps. Transmitter mass did not exceed 2% of the body mass of any individual, which is well within the recommended 3% threshold (Casper, 2009; Soulsbury *et al.*, 2020; Connington *et al.*, 2024). Although transmitters were programmed on various sampling schedules over the study duration, they generally operated on one of two schedules: (i) a high-frequency cycle collecting one GPS fix and an 8-s 20-Hertz ‘burst’ of ACC pathway data (resulting in 160 ACC values) every 4 min, or (ii) a recharge cycle, activated at ~75% battery, collecting one fix and one ACC burst every 1–4 h. Between astronomical dawn and dusk, the transmitter operated on a sleep cycle, where one GPS fix and one burst were taken every 4 h. Movement (GPS) and activity (ACC) data were automatically uploaded from the transmitter via cellular network the Ornitrack manufacturer control panel (https://www.ornitela.com/; https://cpanel.glosendas.net/), and similarly all location-time data were synchronized on the Movebank data repository (https://www.movebank.org/cms/movebank-main; study ID 2741771498). Although our sample of five tracked individuals is small, this is typical for GPS-telemetry studies of large, territorial apex raptors (e.g. Murgatroyd *et al.*, 2018) and our inferences are drawn at the individual level using mixed-effects resource selection function (RSF) frameworks that are explicitly designed to accommodate few individuals with high-resolution location-time data (Muff *et al.*, 2020; Fieberg *et al.*, 2021; Northrup *et al.*, 2022).

### Data analyses

All data preparation and statistical analyses were performed in R, using the RStudio Version 4.4.1 interface (R Core Team, 2024), using data that started 24-h post transmitter deployment and spanned one full year for males, or until the onset of incubation for females. To account for variation in sampling schedules, we standardized the data by retaining only GPS fixes separated by at least 4 min.

Movement (i.e. location-time) and ACC data were imported from Movebank and the Ornitela control panel, respectively, to R, where they were filtered to only include plausible coordinates (within the boundaries of eThekwini Municipality; https://gis-ethekwini.opendata.arcgis.com/). Data were further cleaned by removing duplicate timestamps and fixes that had improbable speed and distance turning angles and, therefore, likely represented erroneous recordings, based on the ‘outlie’ function in the ‘ctmm’ package (Fleming and Calabrese, 2023; Calabrese *et al.*, 2016). With each GPS fix, the transmitter recorded speed, altitude and tag temperature, and an 8-s 20-Hertz ‘burst’ of ACC data. For each ACC burst, we only retained those that contained all 160 ACC values (i.e. removed interrupted burst sequences). We subsequently merged each ACC burst with the values of the corresponding GPS-derived measurements (one value for each of speed, altitude and temperature per ACC burst).

Since crowned eagles are diurnal (Brown and Amadon, 1968), to avoid biases imposed by night roosting behaviour, we only retained daytime fixes using the ‘getsunlighttimes’ function in the package ‘suncalc’ (Thieurmel and Elmarhraoui, 2022) with the time zone set to ‘Africa/Johannesburg’. Additionally, for female birds (*n* = 2; EVE and KEA), we removed all data after the onset of incubation, which was determined by visually inspecting the raw ACC patterns and cross-checking against chick hatch dates [incubation starts 51 days prior to hatch (Steyn, 1983)].

To prepare our data for the resource selection analysis, we first assigned each GPS fix to a corresponding landcover category in the South African National Land Cover (SANLC; https://www.dffe.gov.za/egis) 2022 map (20 × 20 m resolution), using the ‘extract’ function in the ‘terra’ package (Hijmans *et al.*, 2024). We used the coarsest category level, SANLCC_1, which includes nine categories: barren land, built-up, cultivated, forested land, grassland, mines & quarries, shrubland, waterbodies and wetlands. Shrubland was absent in the study area, and so was excluded from our analyses. We also sourced shapefiles from the eThekwini Municipality Open GIS Data portal (https://gis-ethekwini.opendata.arcgis.com/) for the D’MOSS (2018) and for paved roads (2022). We then calculated the distance between each GPS fix and the D’MOSS boundary and nearest road using the ‘nearest’ function in the ‘terra’ package (Hijmans *et al.*, 2024). We used the same method to calculate the distance from each GPS fix to the corresponding bird’s nest (see McPherson *et al.*, 2016a for details on how nests were located). Some birds switched nest location within their territory across the 2 years: the current nest location was used for each datapoint. For individuals that switched nests between 2023 and 2024 breeding seasons, we identified the nest switch date as the first date where >5% of GPS fixes occurred within 30 m of the 2024 nest coordinates.

To quantify energy expenditure, we computed the mean overall dynamic body acceleration (ODBA) following Gleiss *et al.* (2011). First, we isolated static acceleration by grouping data by burst ID and applying a running mean using the ‘rollapply’ function in the ‘zoo’ package (Zeileis and Grothendieck, 2005). We used a 4 s running window in line with other studies of large avian subjects (Soriano-Redondo *et al.*, 2023) and following Shepard *et al.* (2011). We then subtracted this from overall acceleration to determine dynamic acceleration. ODBA was calculated as the sum of the absolute values of dynamic acceleration across all three axes. We then computed the mean ODBA value per ACC burst, such that we could assign the corresponding GPS fix with a single measure of energy expenditure.

### Resource selection

We assessed resource selection on an individual basis, using the package ‘ctmm’ (Fleming and Calabrese, 2023; Calabrese *et al.*, 2016) to fit continuous time movement models to individual telemetry data. Prior to model fitting, we visually inspected the empirical variogram of each individual’s movement data using the ‘variogram’ function to confirm range residency—the assumption that space use is stationary and spatially bounded over the sampling period, which is a prerequisite for valid autocorrelated kernel density estimate (AKDE) estimation (Fleming *et al.*, 2014). Range residency was confirmed in all individuals where the variogram reached a clear asymptote, indicating bounded movement consistent with central place foraging behaviour. Initial models were created using the function ‘ctmm.guess’ and refined using ‘ctmm.select’. From the best-fitting model, we estimated AKDEs using the ‘akde’ function, with home ranges defined at the 95% utilization distribution level. We converted our categorical landcover raster (SANLC map) into a list of factorized rasters and fitted individual RSFs with a Riemann integrator using the ‘rsf.fit’ function.

In this RSF framework, categorical habitat variables must be parameterized with a reference category, which allows the coefficients for other habitat types to be interpreted relative to that baseline (Boyce *et al.*, 2002). Forested land was set as the reference category for all individuals and results were deemed significant if upper and lower 95% confidence intervals (CIs) of parameter estimates did not overlap zero. We selected forest as the reference because it represents the dominant natural habitat in the study area and provides a meaningful ecological baseline. Importantly, the RSF inherently accounts for availability, since used locations are compared to randomly drawn available points within the individual’s accessible area (derived through the individual’s AKDE). Negative or positive coefficients for other habitats therefore indicate that, relative to the area available to the individual, those habitats are used either less or more than forest (Alston *et al.*, 2023). This approach allows formal hypothesis testing, which would not be possible with descriptive indices, making RSFs the standard for broad scale movement ecology analysis (Florko *et al.*, 2025).

### Energy expenditure

Given the right-skewed and non-normal distribution of our mean ODBA response (Supplementary Fig. S2), we used a generalized linear mixed model (GLMM) with a Gamma distribution and a log link to investigate the relationship between mean ODBA and four test predictors: landcover, distance to the nearest road, distance to the D’MOSS boundary, whether the bird was inside or outside of the D’MOSS (hereafter ‘intersection’; a binary predictor with 0 = outside the D’MOSS or 1 = inside the D’MOSS), and the interaction between distance to the D’MOSS and intersection. Landcover categories that were used infrequently (<0.1% of total fixes) were dropped from this analysis to avoid model convergence issues. Our control predictors were distance to the nest, sex and hour of day (as a quadratic term to account for non-linear changes in activity typical of diurnal raptors). Individual and date were included as random effects. We included all theoretically identifiable random slopes in the model to avoid overconfident model estimates and to keep type I error rates at the nominal level of 5% (Schielzeth and Forstmeier, 2009; Barr *et al.*, 2013). Specifically, we included the random slopes of all test and control predictors within date and all predictors excluding sex within individual. The model was fit using the ‘glmmTMB’ function and package (Brooks *et al.*, 2017). We assessed collinearity using the ‘check_collinearity’ function in the ‘performance’ package (Lüdecke *et al.,* 2021) and none was detected.

We began with fitting a maximal model to our response, which included the parameters for the correlations among random slopes and intercepts (Barr *et al.*, 2013). However, the model failed to converge when all correlations were considered. We therefore excluded correlations among random slopes and intercepts from the model, in keeping with current best-practice recommendations for mixed-effects models with limited grouping levels (Bates *et al.*, 2015; Matuschek *et al.*, 2017; Harrison *et al.*, 2018).

To understand whether our test predictors contributed meaningfully to the model, we conducted a full-null model comparison (Forstmeier and Schielzeth, 2011). Here, the null model was specified without the test predictors and with the control predictors. Both the full and null models had the same random effect and slope structure. We then compared the full and null models using a likelihood ratio test (LRT) to evaluate overall model significance ( Dobson and Barnett, 2018 ). Significance in the LRT indicates that the full model with test predictors performs better than the null model without test predictors, and under this condition, we further investigated the influence of the individual predictors.

To assess the overall contribution of each individual predictor in the model, we used the ‘drop1’ function in the ‘stats’ package (R Core Team, 2024), within a null-hypothesis significance testing (NHST) framework (Dobson and Barnett, 2018). Importantly, this does not constitute stepwise model selection: ‘drop1’ evaluates the contribution of each predictor independently by comparing a model lacking the predictor against the full model via LRTs, without sequentially building or reducing a final model. The pre-specified full model is the sole basis for all inference and parameter interpretation. Predictors were considered significant if the resulting *P*-values were <0.05. We pre-specified that, in the event of a non-significant interaction term, a reduced model excluding only that interaction would be fitted to allow unambiguous estimation of the constituent main effects—a standard practice in NHST when interaction and main effects cannot be independently interpreted in the same model (Engqvist, 2005). This step was defined *a priori* and does not represent data-driven model selection; all other predictors remained in the model. We again applied ‘drop1’ to this reduced model to evaluate the main effects. In cases where the drop1 model failed to converge (i.e. when removing landcover), we manually calculated LRT scores using the formula: $\mathrm{LR}=2\times \left(\iota{\ell}_1-{\ell}_2\right)$, where ${\ell}_1$and ${\ell}_2$ are the log-likelihoods of the full and reduced model, respectively (Lewis *et al.*, 2011). We cross-checked this manual approach by repeating it across predictors from the ‘drop1’ analysis that did converge. We interpret our model output in the context of both the significance of individual predictors as assessed by the ‘drop1’ NHST approach and the effect sizes and fitted values derived from the full (or *a priori*-reduced) model. At no point were non-significant predictors removed to modify the model for inference. We plotted significant effects using ‘ggplot2’ (Wickham, 2016), and extracted predictions from the model using the ‘predict’ function (R Core Team, 2024). Covariates were z-transformed for modelling and back-transformed for plotting and visualization.

### Ethics and permits

The capture and fitting of telemetry were performed under the permits: Ezemvelo KZN Wildlife (OP3005 and OP3706) and University of KwaZulu-Natal Animal Research Ethics Council (AREC/00006155).

## Results

### Resource selection

**Figure 1:**
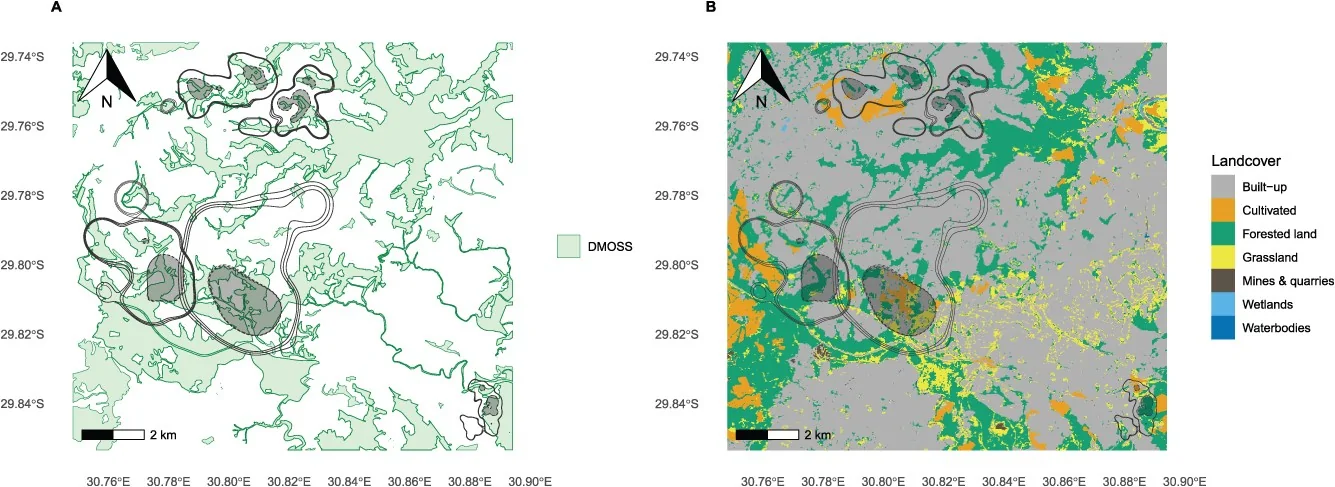
Space use of crowned eagles (*Stephanoaetus coronatus*; *n* = 5) in Durban, South Africa, shown against **(a)** the D’MOSS and **(b)** landcover categories determined by the 2022 South African National Land Cover map. Dashed grey polygons with shading indicate 50% AKDEs (core areas), while the triple solid grey lines show 95% AKDEs: the middle line is the estimate boundary, the outer lines represent the lower and upper confidence bounds

Individual resource selection models from 5-tagged breeding adults analysed three males: ACE (9237 observations), ADM (37 819 observations) and STV (32 542 observations), and two females: EVE (7034 observations) and KEA (17 783 observations). Space-use, estimated using 95% AKDE, varied considerably between individuals (Supplementary Table S3). The mean 95% AKDE was 6.794 km^2^. There was a 10-fold difference in the range size between the individual with the smallest home range (ADM, estimate: 1.434 km^2^; 95% CI lower to upper bounds: 1.382–1.488 km^2^) and that with the largest (ACE, 16.437 km^2^; 14.755–18.208 km^2^). Home range sizes for the other individuals were as follows: STV (4.505 km^2^; 4.271–4.746 km^2^), EVE (2.820 km^2^; 2.610–3.038 km^2^) and KEA (8.772 km^2^; 8.011–9.567 km^2^). Two individuals showed a small degree of spatial overlap between their AKDEs (KEA and ACE; Fig. 1).

Across individuals, built-up habitat was consistently less selected relative to forested land, given the area available to the individual (as defined by the AKDE), with RSF estimates ranging from −1.480 to −2.706 (Supplementary Table S4; 95% CI lower—upper bounds across individuals: ACE: −1.720 to −1.239; ADM: −2.333 to −2.101; EVE: −1.939 to −1.621; KEA: −2.349 to −1.881; STV: −2.830 to −2.582). Some individuals also exhibited strong avoidance of grassland and cultivated habitats, relative to forested land, although there was no consistent pattern between individuals [e.g. grassland: ACE = −1.557 (−2.109 to −1.005); ADM = −1.936 (−2.150 to −1.723); KEA = −1.849 (−2.577 to −1.121); STV = −4.572 (−6.815 to −2.329); and cultivated: ACE = −0.425 (−0.780 to −0.069); ADM = −0.388 (−0.533 to −0.243); KEA = −1.897 (−2.533 to −1.241); STV = −3.298 (−3.551 to 3.045)]. All other landcover categories showed no significant difference relative to forest. Categories with very few fixes (particularly mines & quarries, wetlands and waterbodies) had high variance in RSF estimates, resulting in wide CIs and unstable coefficients, and reflecting the low sample size rather than biological avoidance or preference (Supplementary Table S4).

### Energy expenditure

**Figure 2:**
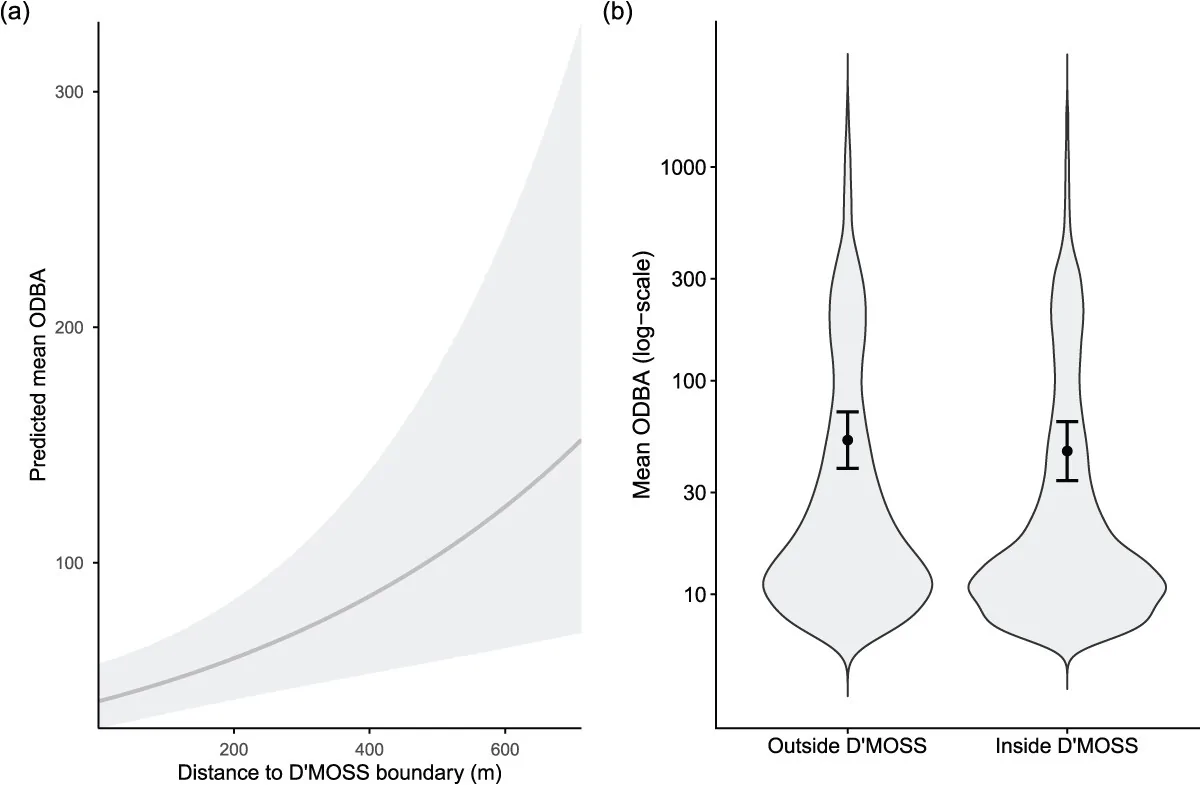
**(a)** The relationship between mean ODBA and distance to the Durban Metropolitan Open Space (D’MOSS) boundary in crowned eagles (*Stephanoaetus coronatus*; *n* = 5 individuals; *n* = 144 871 observations). The solid line represents model predictions from a GLMM with a Gamma distribution and a log link. The shaded ribbon indicates 95% upper and lower CIs. **(b)** Distribution of raw mean ODBA values inside and outside the D’MOSS boundaries, shown as violin plots. Black points represent model-predicted overall mean ODBA, with error bars indicating 95% upper and lower CIs from the same GLMM. Predictions and raw data are presented on a logarithmic scale to aid visualization

**Figure 3:**
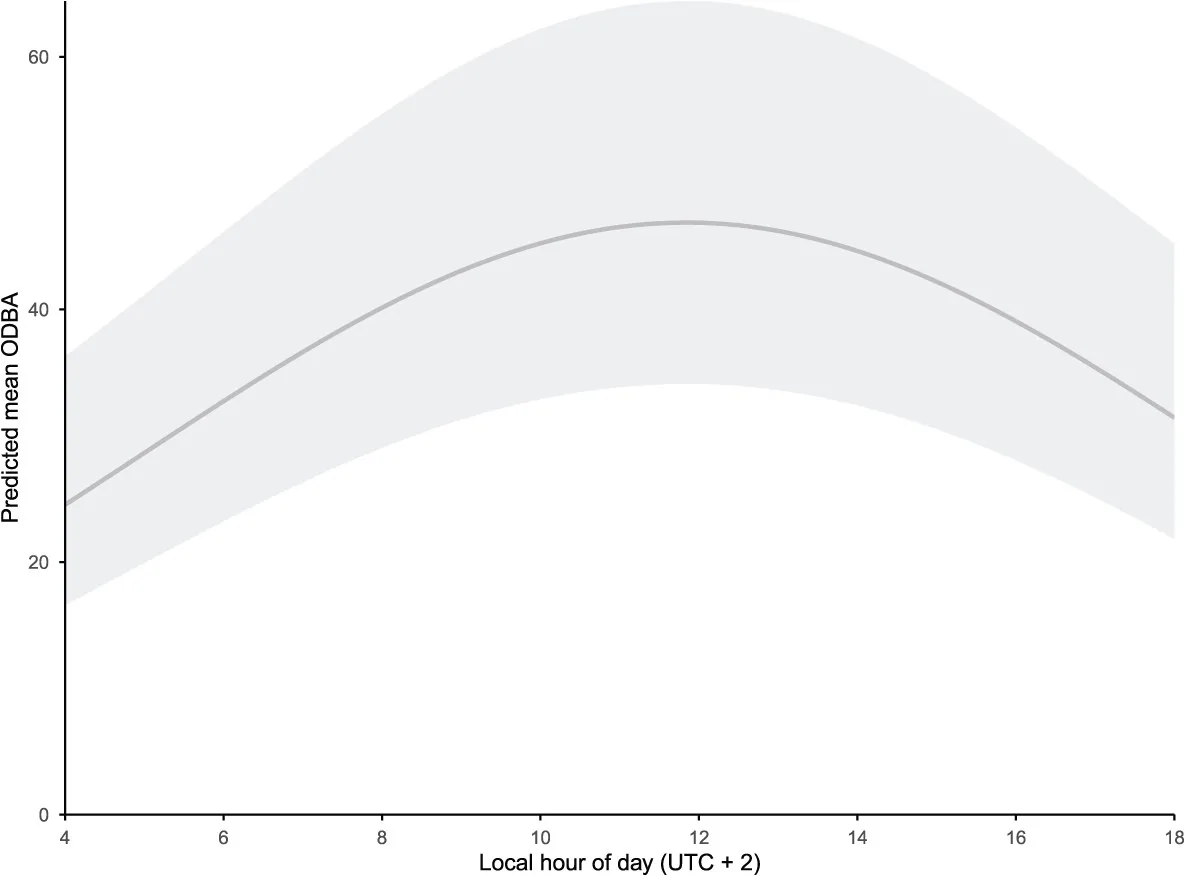
Relationship between mean ODBA and local hour of day (*n* = 144 871 observations) in an urban population of crowned eagles (*S. coronatus*; *n* = 5 individuals). The solid line represents model predictions from a GLMM with a Gamma distribution and log link. The shaded ribbon indicates 95% upper and lower CIs

We used 144 871 observations from the five crowned eagles to investigate energy expenditure of crowned eagles in an urban-forest mosaic landscape (Supplementary Table S5). The null model vs full model comparison was significant (*P* < 0.05; Supplementary Table S6), demonstrating that the predictors collectively contributed significant explanatory power. ‘Drop1’ revealed that the interaction term between D’MOSS intersection and distance to the D’MOSS boundary was not significant (Supplementary Table S7). As pre-specified, the non-significant interaction term was excluded from the model to allow independent estimation of the constituent main effects. Applying ‘drop1’ to this *a priori*-reduced model, distance from the D’MOSS boundary was a significant predictor of mean ODBA (drop1: *P* = 0.015, Supplementary Table S8). Model coefficients from the *a priori*-reduced model indicate higher energy expenditure with increasing distance from the D’MOSS boundary in either direction (deeper into the D’MOSS, or further away from it, Fig. 2a; Table 1). Intersection did not reach significance at the 0.05 threshold (drop1: *P* = 0.062; Supplementary Table S6); however, model coefficients indicated that energy expenditure was higher outside of the D’MOSS (Table 1; Fig. 3). Distance to the nearest road was non-significant (Supplementary Table S8 and Supplementary Fig. S4a). Manual calculation of LRT scores indicated that landcover was also non-significant as an overall predictor (drop1: *P* = 0.116, Supplementary Table S8), although model coefficients indicated that ODBA in built-up areas was significantly higher relative to forested land (Table 1: *P* = 0.024; Supplementary Fig. S3). Among the control predictors, time of day as a quadratic term was highly significant (drop1: *P* < 0.001; Supplementary Table S8), with model coefficients increasing mean ODBA from the early morning to peak around 11 a.m., and slowly decreasing through the afternoon (Fig. 3). Distance to nest (Supplementary Fig. S4b) and sex (Supplementary Fig. S5) were non-significant.

**Table 1:** Model output summary from a GLMM analysing the mean ODBA of crowned eagles (*S. coronatus*; *n* = 5 individuals; *n* = 144 871 observations) in Durban, South Africa

**Parameter**	**Lower CI**	**Estimate**	**Upper CI**	**SE**	***P*-value**
**Intercept**	**3.661**	**3.965**	**4.268**	**0.155**	**<0.001**
**Urban built-up**	**0.015**	**0.119**	**0.223**	**0.053**	**0.024**
Cultivated	−0.031	0.176	0.384	0.106	0.096
Grassland	−0.124	−0.010	0.105	0.059	0.870
**Distance to D’MOSS**	**0.069**	**0.171**	**0.273**	**0.052**	**0.001**
**Intersection (inside D’MOSS)**	**−0.225**	**−0.118**	**−0.012**	**0.054**	**0.029**
Distance to road	−0.117	0.000	0.117	0.059	0.998
Distance to nest	−0.103	−0.030	0.044	0.037	0.425
Sex (female)	−0.0191	0.269	0.657	0.198	0.174
Hour	0.014	0.026	0.668	0.021	0.203
**Hour^2**	**−0.181**	**−0.133**	**−0.085**	**0.025**	**<0.001**

The model was fitted with a Gamma distribution and log link and included individual and date as random effects. Fixed effects included landcover type, distance to D’MOSS boundary, D’MOSS intersection (a binary variable where 0 = outside and 1 = inside), distance to nearest road, distance to the individual’s nest, sex and hour of day (included as a quadratic term). Individual and date were included as random effects. Reference levels for categorical variables were ‘Forest’ for landcover type, ‘Outside D’MOSS’ for intersection, and ‘Male’ for sex. Significant predictors are shown in bold.

## Discussion

Our investigation into individual resource use and energy expenditure in crowned eagles reveals some of the physiological consequences of navigating a heterogeneous urban-forest landscape. As predicted, resource selection analyses showed that eagles consistently selected forests over built-up habitats, relative to availability. In addition, built-up areas were associated with higher energy expenditure compared to forests, suggesting that highly urban areas impose greater energy expenditure. Although overall energy expenditure was higher outside the D’MOSS than inside, it also increased with distance from the D’MOSS boundary, regardless of whether the bird was located inside or outside the D’MOSS. Given that the D’MOSS, despite encompassing ecologically viable green space in the city, is also highly heterogeneous and interspersed with anthropogenic developments, we suggest that the varied opportunities and pressures both inside and outside the D’MOSS area can influence the eagles’ energetic demands. These findings extend growing evidence that integrates GPS tracking with ACC to provide a uniquely powerful approach to examine energetic consequences of habitat use in predators navigating human-modified landscapes (English *et al.*, 2024; Ellis-Soto *et al.*, 2025).

The crowned eagles’ relative preference of forested over built-up habitat, alongside the associated energy expenditure patterns in these habitats, builds on previous work showing that this population heavily relies on forested land (McPherson *et al.*, 2016a, 2019; Maseko *et al.*, 2023). While high- and low-energy activities occur across both habitat types, we find that the physiological costs imposed by the urban environment are greater than that of forested areas. Since crowned eagles in Durban experience human–wildlife conflict and are at risk of direct persecution (McPherson *et al.*, 2021), eagles navigating urban habitats may remain in a more alert or active moving state to minimize the risk of encountering people. Urban habitats also come with risks from road collisions and human infrastructure (e.g. electrocution), evidenced by the fact that two of the five individuals in this study have since died due to suspected road (ADM) and confirmed window collisions (STV; unpublished data). Furthermore, urban areas may also provide fewer suitable structures for low-energy activities (i.e. perching and roosting), potentially increasing movement complexity and costly flight responses away from risks and between patches of suitable habitat.

The costs imposed by anthropogenic disturbance and altered urban landscapes, including those mediated through landscapes of fear, are supported by a growing body of quantitative literature (Ciuti *et al.*, 2012;  Zanette and Clinchy, 2019; Gaynor *et al.*, 2021). Research analysing large carnivore movement data shows an increase in movement speed with increasing proximity to towns and trails—areas assumed to have higher levels of human activity and densities of infrastructure (Whittington *et al.*, 2022). Evidence from ACC-equipped pumas (*Puma concolor*) demonstrates that individuals expend ~13% more calories per 5-min period in areas near human development, compared to wildland habitats of equivalent terrain (Nickel *et al.*, 2021). Tigers (*Panthera tigris*) showed similarly elevated activity costs in areas of high anthropogenic disturbance (Hussain *et al.*, 2026), and barred owls (*Strix varia*) inhabiting structurally degraded urban fragments showed higher nocturnal energy expenditure than individuals in high-quality forested patches (Jirinec *et al.*, 2024). Although the landscape-of-fear framework has thus far been predominantly applied to terrestrial predators, including large urban carnivores, some evidence suggests analogous physiological costs in aerial predators. Taken together, the landscape of fear and structural costs for crowned eagles and other large urban raptors are likely expressed less as elongated foraging paths and more as forced flapping flight where thermal subsidy is weak above built-up matrix (Shepard *et al.*, 2013; Sage *et al.*, 2022), elevated detectability in built-up urban habitat with suspected consequences for direct persecution (McPherson *et al.*, 2021), and increased flight-initiation distances near humans (Møller, 2008). We note that not all forms of flight are energetically equivalent for all large-bodied raptors: flapping flight is several-fold more costly than gliding or thermal soaring (Norberg, 1996; Shepard *et al.*, 2013; Duriez *et al.,* 2014). Because crowned eagles are short-winged, high-wing-loading forest predators that are mechanically ill-suited to sustained soaring (Pennycuick, 1989), most flight beyond the canopy is likely to be flapping or short-glide flight (Supplementary Tables S1 and S2)—precisely the flight modes for which ODBA most closely tracks metabolic rate (Halsey *et al.*, 2009; Sakamoto *et al.*, 2009). This reinforces our interpretation of elevated ODBA in built-up areas as a real energetic cost but may not apply in the same manner to other large-bodied raptors. Nevertheless, we acknowledge that our ODBA-based estimates do not explicitly account for all non-flapping flight behaviours. Although soaring and extended gliding represented only a relatively small proportion of observed movement (~15%), these flight modes can reduce mechanical body acceleration while aerodynamic costs remain non-zero, potentially weakening the relationship between ODBA and true metabolic expenditure (Duriez *et al.*, 2014; Wilson *et al.*, 2020; Conners *et al.*, 2024). We therefore interpret ODBA as a robust proxy for relative energetic expenditure across habitats, while recognizing that absolute energetic costs associated with occasional soaring or gliding may be modestly underestimated. Given the predominantly flapping flight behaviour expected for crowned eagles, we do not consider this limitation sufficient to alter the overall ecological patterns reported here.

Given that habitat outside of D’MOSS boundaries was predominantly urban (88% urban landcover within the 1.05× space-use extent), the landscape of fear and structural costs plausibly explain the higher ODBA values we observed occurring outside D’MOSS boundaries, and deeper into the urban matrix away from the boundaries. In line with our expectations, we also found that deeper into the D’MOSS, energy expenditure increased. We propose that this trend is driven by increased hunting effort within the D’MOSS. Since crowned eagles are ambush hunters that rely on startling their prey (Shultz and Thomsett, 2007), they may avoid hunting in increasingly urban areas where human disturbance factors affect predator and prey alike, with both expected to become more elusive as urbanization intensifies (Gaynor *et al.*, 2018). This avoidance dynamic has recently been formalized as the ‘human shield hypothesis’, whereby predator aversion to areas of high human disturbance effectively creates spatial refugia for prey—further constraining where apex predators can forage profitably (Gaynor *et al.*, 2025). In contrast, prey deeper within the D’MOSS core may be more likely to be slow-moving or at rest, in line with recent research showing that predation events in urban green spaces were higher closer to core areas, with moderate anthropogenic disturbance at habitat edges deterring predators (Wang *et al.*, 2025). Increased energy expenditure with greater distance from the D’MOSS boundary in both directions suggests that the D’MOSS boundary represents a functional threshold where the dominant energy costs shift from foraging effort within to disturbance avoidance outside.

While forested land is conserved within the D’MOSS network, landcover types across the landscape are highly heterogenous. In our system, 91% of fixes in forested land occurred within the D’MOSS boundaries, compared to 40% of fixes in built-up land. This habitat heterogeneity captured within the D’MOSS boundaries highlights the importance of considering broader landscape structure beyond habitat type alone (Frey *et al.*, 2018). In other words, spatial planning frameworks that partition areas dedicated to biodiversity conservation can shape how species experience the same landcover type within such a framework vs outside of one.

Our results showed no significant relationship between the distance to the nearest road and energy expenditure. This may partially further reflect habitat heterogeneity but may also be a limitation of the municipal dataset, since it does not distinguish between paved road types of varying traffic intensities (i.e. major roads vs residential streets). Nonetheless, this finding is consistent with previous studies that demonstrate crowned eagles in Durban show no apparent aversion to roads when selecting nest sites (McPherson *et al.*, 2016a; Maseko *et al.*, 2024), demonstrating at least partial tolerance. This contrasts patterns reported in other large-bodied raptors. An analysis of Mediterranean raptor communities show species-specific responses to roads, with traffic volume being a central driver of foraging habitat selection. Opportunistic species, such as kites, increase their use of areas near roads (Catitti *et al.*, 2025), while large-bodied species tend to avoid them, particularly at high traffic volumes (Planillo *et al.*, 2015). These patterns highlight that road effects are context- and species-specific, but for crowned eagles navigating Durban, despite no apparent relationship with ODBA, roads impose severe indirect costs through collision risk, which disproportionately affects raptors occupying urban environments (McPherson *et al.,* 2016a; Bullock *et al.,* 2024).

Beyond spatial variation, our results also suggest that temporal factors influence energy expenditure. Hour of the day, included as a control in the model to account for diurnal activity patterns, was a significant predictor of ODBA and peaked at 11 a.m., before decreasing slowly into the afternoon. This morning peak in energy expenditure likely reflects the combined influence of multiple variables: thermoregulatory constraints, optimal flight conditions, prey availability and human activity cycles (Lang *et al.*, 2019; Perona *et al.*, 2019; Green *et al.*, 2022; Jain *et al.*, 2026). For large-bodied birds in particular, they must also use the landscape and favourable environmental conditions in a manner that supports energetically costly flight (Shepard *et al.*, 2011; Lanzone *et al.*, 2012). Thermal soaring is one mechanism that may contribute to this pattern, but it can result in a decoupling between ODBA and true energetic cost (Duriez *et al.*, 2014; Conners *et al.*, 2024). As discussed above, we expect this effect to be limited in crowned eagles because of their morphology and flight behaviour. More relevant to the observed late morning peak is the tendency of diurnal raptors to concentrate hunting during morning hours, to avoid thermal stress while exploiting peak prey activity (Rijnsdorp *et al.*, 1981; Roth and Lima, 2007; Tate and Amar, 2017; Naves-Alegre *et al.*, 2025). The relatively late morning peak for crowned eagles (11 a.m.; Fig. 3) may be partially influenced by the forest habitats they occupy, where dense tree cover can buffer thermal extremes and maintain cooler microclimates (De Frenne *et al.*, 2019) that could support hunting activity into the later morning, despite increasing ambient temperatures. Additionally, prey species often shift their activity patterns to avoid predation and anthropogenic disturbance, creating intricate temporal landscapes where predators must balance hunting opportunities against human activity cycles (Lewis *et al.*, 2021; Van Scoyoc *et al.*, 2023).

We acknowledge our study has a limited sample size (*n* = 5); however, this is consistent with other examples of raptor research that achieve detailed results from high-resolution movement data with small sample sizes (Murgatroyd *et al.*, 2016; Carter *et al.*, 2019; Zvidzai *et al.*, 2022). It is also important to note that when using individual-level RSFs, statistical power depends primarily on the number of relocation observations, as opposed to the total number of animals (Northrup *et al.*, 2022). We do not present population-level conclusions, but rather highlight the consistent trends of individual crowned eagles exploiting an anthropogenic environment. This study also demonstrates the promise of biologging to integrate behaviour with physiology, to investigate the potential mechanisms that allow even specialized, large-bodied predators to navigate diverse urban landscapes—provided that sufficiently connected natural habitat is available. Crowned eagles in Durban are moving through a heterogeneous environment with varying degrees of protection. In this environment, they experience energetic expenditure in ways that can be explained by the respective opportunities and costs presented across the urban-forest mosaic landscape. From a conservation physiology perspective, these landscape-driven differences in ODBA are not merely behavioural observations; sustained elevations in metabolic expenditure can deplete energy reserves, compromise body condition and ultimately reduce reproductive investment and survival (Wikelski and Cooke, 2006; Cooke *et al.*, 2013). By mapping where and when elevated energetic costs occur within the urban mosaic, biologging studies such as ours can directly inform urban planning decisions by highlighting habitat features that are physiologically costly to navigate and prioritizing their mitigation or formal protection within green space networks (Ellis-Soto *et al.*, 2025). We therefore suggest that these findings should be applied to encourage urban planning strategies that maximize the formal protection of natural habitat that supports behaviour and physiology, to best sustain wildlife in cities.

## Supplementary Material

Web_Material_coag068

## Data Availability

The processed data and code from one individual (STV) are published on Zenodo for our analyses to be reproduced: https://doi.org/10.5281/zenodo.21887263. The raw movement and ACC datasets will be made available upon request via www.movebank.org ID 2741771498 to conserve sensitive information on nesting sites.
