# Supplementary material for: Resource use and associated energy expenditure of African crowned eagles (Stephanoaetus coronatus) in an urban-forest mosaic landscape
Source: Conserv Physiol. 2026 Sep 18;14(1):coag068. doi: 10.1093/conphys/coag068 (PMC13588711; doi:10.1093/conphys/coag068)
Supplement: Supplementary file 1 [file web_material_coag068.docx]

**Supplementary Materials**

**Resource use and associated energy expenditure of African crowned eagles (*Stephanoaetus coronatus*) in an urban-forest mosaic landscape**

Lara Howard^1,2‡^, Varalika Jain^2,3,‡*^, Shane C Sumasgutner^2,4^, Jesús Bautista^5^, Ralph Buij^6,7^, Colleen T Downs^4^, Susan J Cunningham^1^, Petra Sumasgutner^1,2,3*^

^1^ *FitzPatrick Institute of African Ornithology, Department of Biological Sciences, University of Cape Town, Rondebosch, 7701, Cape Town, South Africa*

^2^ *Konrad Lorenz Research Center for Behavior and Cognition, Core Facility of the University of Vienna, Grünau im Almtal, 4645, Austria*

*^3^ Department of Behavioral and Cognitive Biology, University of Vienna, Vienna, 1030,* *Austria*

*^4^ Centre for Functional Biodiversity, School of Life Sciences, University of KwaZulu-Natal, Scottsville, 3209, Pietermaritzburg, South Africa*

*^5^ Agencia de Medio Ambiente y Agua, Consejería de Sostenibilidad, Medio Ambiente y Economía Azul, Junta de Andalucía, Sevilla, Spain*

*^6^ Animal Ecology Group (AEG), Wageningen Environmental Research, P.O. Box 47,*

*6700 AA Wageningen, The Netherlands*

*^7^ The Peregrine Fund, Boise, ID, USA*

^‡^ Contributed equally, shared first authorship

* Corresponding Authors: [varalika.jain@univie.ac.at](mailto:varalika.jain@univie.ac.at) & [petra.sumasgutner@univie.ac.at](mailto:petra.sumasgutner@univie.ac.at)

**Supplementary Tables**

**Table S1**: Summary of speed-based GPS fix classifications (n = 26,690) as stationary (<3 m/s) or moving (≥3 m/s) for 5 male and 5 female crowned eagles, recorded at 4-minute intervals during daylight hours only over a 15-day period between 08 and 23 May 2026 in Durban, South Africa. Each fix was assumed to represent 4 minutes of moving or stationary behaviour, from which total and average daily (mins/day) time budgets were derived. Across 10 individuals, moving behaviour (i.e., flight) ranged from 1.2-6.4% of fixes (mean 4.3%), equating to 8-47 mins/day (mean 30.3 mins/day).

| **Individual** | **Sex** | **Time stationary (mins)** | **Time moving (mins)** | **Average time stationary p/day (mins)** | **Average time moving p/day (mins)** | **Proportion of GPS fixes moving (%)** |
| --- | --- | --- | --- | --- | --- | --- |
| RYE  QUB | Female  Female | 10100  10220 | 696  576 | 673.3  681.3 | 46.4  38.4 | 6.4  5.3 |
| WAV  POI | Female  Female | 10540  10392 | 256  408 | 702.7  692.8 | 17.1  27.2 | 2.4  3.8 |
| HEX | Female | 10328 | 364 | 688.5 | 24.3 | 3.4 |
| MAC | Male | 10216 | 584 | 681.1 | 38.9 | 5.4 |
| IWI | Male | 10264 | 532 | 684.3 | 35.5 | 4.9 |
| BUG | Male | 10340 | 416 | 689.3 | 27.7 | 3.9 |
| OWL | Male | 10076 | 588 | 671.7 | 39.2 | 5.5 |
| MP3 | Male | 9744 | 120 | 649.6 | 8.0 | 1.2 |

**Table S2**: Estimated daily thermal soaring time for one adult male crowned eagle, based on two multi-day periods when the transmitter's 'Flight Detection' mode was active (Period A: 22 days; Period B: 61 days). Flight types (e.g., thermal soaring) were distinguished using raw accelerometry, GPS altitude, and heading-rate traces (see Supplementary Figure S6). For each period, daily soaring time was calculated by multiplying the mean daily duration of Flight Detection activity by the mean proportion of that flight time classified as thermal soaring. This gave an estimated soaring budget of 3-5 minutes per day, or ~0.5-0.7% of a 12-hour daytime period.

|  | **Period A (22 days)** | **Period B (61 days)** |
| --- | --- | --- |
| Mean daily time in ‘Flight Detection’ mode (mins) | 35 | 27 |
| Estimated daily thermal soaring time (mins) | 5.06 | 3.44 |
| Mean daily thermal soaring (% of flight time) | 14.46 | 12.74 |
| **% soaring of 12-hour daily activity period** | **0.70** | **0.47** |

**Table S3**: Space use estimates (individual AKDEs) and the corresponding upper and lower confidence intervals for crowned eagles (*Stephanoaetus coronatus*; *n* = 5) across greater Durban, South Africa. The number of observations varied by individual due to occasional differences in sampling schedules and, for female birds (EVE and KEA), the onset of incubation: ACE, *n*=9,237; ADM, *n*=37,819; STV, *n*=32,542; EVE, *n*=7,034; and KEA, *n*=17,783. Values are shown in km^2^. Note: ACE was in the process of switching territories.

| **Individual** | **Sex** | **Lower CI** | **Estimate** | **Upper CI** |
| --- | --- | --- | --- | --- |
| ACE | M | 14.755 | 16.437 | 18.208 |
| ADM | M | 1.382 | 1.434 | 1.488 |
| STV | M | 4.271 | 4.505 | 4.746 |
| EVE | F | 2.610 | 2.820 | 3.038 |
| KEA | F | 8.011 | 8.772 | 9.567 |

**Table S4**: Individual-level resource selection function (RSF) outputs for crowned eagles (*Stephanoaetus coronatus*; *n* = 5) in Durban, South Africa. Model estimates and corresponding 95% confidence intervals (CIs) are presented for each individual across landcover categories derived from the 2022 South African National Landcover map. Outputs are interpreted relative to forested land, which was the reference category across all individuals. Wide CIs in some categories indicate low sample sizes (i.e., few GPS fixes in those categories), but were retained in the models as they reflect available habitat. Significant results are those that did not overlap zero, and are shown in bold.

| **Individual** | **Landcover** | **Lower CI** | **Estimate** | **Upper CI** |
| --- | --- | --- | --- | --- |
| **ACE** | Wetlands | -4.074 | -0.160 | 3.754 |
|  | Waterbodies | -595.035 | -9.474 | 576.087 |
|  | Mines & quarries | -464.060 | -8.246 | 447.569 |
|  | **Grassland** | **-2.109** | **-1.557** | **-1.005** |
|  | **Cultivated** | **-0.780** | **-0.425** | **-0.069** |
|  | **Built-up** | **-1.720** | **-1.480** | **-1.239** |
|  | Barren land | -9.719 | -2.642 | 4.435 |
| **ADM** | Wetlands | -25.357 | -1.765 | 21.827 |
|  | Waterbodies | <-1000 | 0.104 | >1000 |
|  | Mines & quarries | <-1000 | -0.249 | >1000 |
|  | **Grassland** | **-2.150** | **-1.936** | **-1.723** |
|  | **Cultivated** | **-0.533** | **-0.388** | **-0.243** |
|  | **Built-up** | **-2.333** | **-2.218** | **-2.101** |
|  | Barren land | -2.229 | -0.755 | 0.719 |
| **EVE** | Wetlands | -111.844 | -5.637 | 100.570 |
|  | Waterbodies | <-1000 | 1.757 | >1000 |
|  | Mines & quarries | <-1000 | -0.431 | >1000 |
|  | Grassland | -9.054 | -4.267 | 0.520 |
|  | Cultivated | <-1000 | -1.856 | >1000 |
|  | **Built-up** | **-1.939** | **-1.780** | **-1.621** |
|  | Barren land | -506.149 | -8.119 | 489.912 |
| **KEA** | Wetlands | -3923.668 | -9.887 | 3903.893 |
|  | Waterbodies | -13.011 | -4.282 | 4.446 |
|  | Mines & quarries | -307.831 | -6.701 | 294.429 |
|  | **Grassland** | **-2.577** | **-1.849** | **-1.121** |
|  | **Cultivated** | **-2.553** | **-1.897** | **-1.241** |
|  | **Built-up** | **-2.349** | **-2.115** | **-1.881** |
|  | Barren land | -138.801 | 11.492 | 12.549 |
| **STV** | Wetlands | -307.020 | -10.659 | 285.703 |
|  | Waterbodies | -18.859 | -6.435 | 5.988 |
|  | Mines & quarries | <-1000 | 0.096 | >1000 |
|  | **Grassland** | **-6.815** | **-4.572** | **-2.329** |
|  | **Cultivated** | **-3.551** | **-3.298** | **3.045** |
|  | **Built-up** | **-2.830** | **-2.706** | **-2.582** |
|  | Barren land | -13.456 | -5.157 | 3.141 |

**Table S5:** Distribution of GPS fixes (*n*=144, 871) from crowned eagles (*n*=5; *Stephanoaetus coronatus*) across landcover categories. Landcover categories with limited data (<0.1% of fixes) were dropped from analysis. The table shows the total number of fixes per landcover type alongside the corresponding number and proportion that occurred within the boundaries of the Durban Metropolitan Open Space System (D’MOSS).

| **Landcover category** | **No. of total fixes** | **No. of fixes inside D’MOSS** | **Proportion of fixes inside D’MOSS** |
| --- | --- | --- | --- |
| Forested land | 96141 | 87578 | 0.911 |
| Built-up | 38903 | 15743 | 0.405 |
| Cultivated | 7487 | 5429 | 0.725 |
| Grassland | 2340 | 1425 | 0.609 |

**Table S6:** Likelihood ratio test (LRT) results comparing a null model to a full generalised linear mixed model (GLMM) with a Gamma distribution and a log link, fitted to crowned eagle (*Stephanoaetus coronatus*; *n*=5) biologging data. The null model included three control parameters: distance to nest, hour of day, and sex. The full model retained these controls, while additionally included four predictor variables of interest: landcover category, distance to the nearest road, distance to the Durban Metropolitan Open Space System (D’MOSS) boundary, and D'MOSS intersection (a binary variable where 0=outside and 1=inside), with an interaction term between distance to the D'MOSS boundary and D'MOSS intersection.

|  | **AIC** | **BIC** | **logLiik** | **Chisq** | **df** | **p value:** |
| --- | --- | --- | --- | --- | --- | --- |
| Null model (control parameters) | 1436375 | 1436661 | -718158.3 |  | 29 |  |
| Full model | 1436372 | 1436728 | -718150 | 16.475 | 36 | 0.021 |

**Table S7:** ‘drop1’ results for the full generalised linear mixed model (GLMM) fitted with a Gamma distribution and a log link to crowned eagle (*Stephanoaetus coronatus*; *n* = 5) GPS and accelerometery data (*n*=144,871 observations). The model included three control variables: distance to nest, hour of day, and sex, and four predictors: landcover category, distance to nearest road, distance to the Durban Metropolitan Open Space System (D’MOSS) boundary, and D’MOSS intersection, a binary variable where 0 = outside and 1 = inside). The model included an interaction term between distance to the D’MOSS boundary and D’MOSS intersection. Each row shows the effect of removing a single term from the model, with associated change in AIC, likelihood ratio χ² statistic, degrees of freedom, and p-value. Significant results are shown in bold.

|  | **AIC** | **chisq:** | **df:** | **p value:** |
| --- | --- | --- | --- | --- |
| <none> | 1436372 |  |  |  |
| Landcover | 1436372 | 5.749 | 3 | 0.124 |
| Distance to road | 1436370 | 0.000 | 1 | 0.996 |
| Distance to nest | 1436371 | 0.608 | 1 | 0.436 |
| Sex | 1436372 | 1.566 | 1 | 0.211 |
| Hour | 1436372 | 1.534 | 1 | 0.216 |
| **Hour^2** | **1436381** | **11.101** | **1** | **0.001** |
| Distance to D’MOSS*intersection | 1436371 | 1.001 | 1 | 0.317 |

**Table S8:** ‘drop1’ results for the a priori-reduced generalised linear mixed model (GLMM) fitted with a Gamma distribution and a log link to crowned eagle (*Stephanoaetus coronatus*; *n* = 5) GPS and accelerometery data (*n*=144,871 observations). The model included three control variables: distance to nest, hour of day, and sex, and four predictors: landcover category, distance to nearest road, distance to the Durban Metropolitan Open Space System (D’MOSS) boundary, and D’MOSS intersection, a binary variable where 0 = outside and 1 = inside). This model excluded the interaction term between distance to D’MOSS boundary and intersection. Each row shows the effect of removing a single term from the model, with associated change in AIC, likelihood ratio χ² statistic, degrees of freedom, and p-value. Manual calculation of landcover LRT scores showed as non-significant (*p*=0.116). Significant results are shown in bold.

|  | **AIC** | **chisq:** | **df:** | **p value:** |
| --- | --- | --- | --- | --- |
| <none> | 1436372 |  |  |  |
| Landcover | - | - | 3 | - |
| **Distance to D’MOSS** | **1436375** | **5.961** | **1** | **0.015** |
| Intersection | 1436373 | 3.489 | 1 | 0.062 |
| Distance to road | 1436369 | 0.000 | 1 | 0.990 |
| Distance to nest | 1436370 | 0.697 | 1 | 0.436 |
| Sex | 1436371 | 1.579 | 1 | 0.209 |
| Hour | 1436371 | 1.535 | 1 | 0.215 |
| **Hour^2** | **1436380** | **11.103** | **1** | **0.001** |

**Supplementary Figures**

**Figure S1:** Locations of crowned eagle nests (*n*=5) in the Durban, South Africa, during the 2023 and 2024 breeding seasons. Nest sites are colour coded by individual, with boxes corresponding to 2023 nests and circles to 2024 nests. The Durban Metropolitan Open Space System (D’MOSS) is showed in green fill, and the road network in grey. Note: Individuals ADM and EVE retained the same nests across both years.

**Figure S2:** Frequency distribution of mean Overall Dynamic Body Acceleration (ODBA) across all GPS fixes in our study of crowned eagles in Durban, South Africa (*Stephanoaetus coronatus*; *n*= 5 individuals; *n*=144,871 observations). Data are highly right-skewed, reflecting predominantly low-energy observations recorded by high-resolution sampling.

**Figure S3:** The relationship between mean Overall Dynamic Body Acceleration (ODBA) and landcover type in an urban population of crowned eagles (*Stephanoaetus coronatus*; *n*=5 individuals; *n*=144,871 observations). Model predictions are from a generalised linear mixed model with a Gamma distribution and log link. Mean model estimates are shown as black circles and 95% upper and lower confidence intervals as error bars. Raw data are displayed as scatter points showing (a) the full distribution and (b) values below the 95th percentile to improve visibility of model predictions.

**Figure S4:** The relationship between mean Overall Body Dynamic Acceleration (ODBA) and distance in metres to (a) the nearest road and (b) the individual’s nest in a population of crowned eagles (*Stephanoaetus coronatus*; *n*=5) in Durban, South Africa. Model predictions are from a generalised linear mixed model with a Gamma distribution and a log link. The mean model predictions are indicated by the solid grey line, upper and lower 95% confidence intervals are shown in the shaded ribbon.

**Figure S5**: Distribution of raw mean Overall Body Dynamic Acceleration (ODBA; *n*=144,871 observations) in crowned eagles (*Stephanoaetus coronatus*; *n*=5) by sex, shown as violin plots. Model predictions are from a generalised linear mixed model with a Gamma distribution and a log link. The black points represent model-predicted overall mean ODBA, with error bars indicating 95% upper and lower confidence intervals. Data are presented on a logarithmic scale to aid visualisation.

**Figure S6**: Example flight segment from Flight Detection data analysis to distinguish flight behaviours through raw accelerometry (xyz), GPS altitude, heading and magnetic (xyz) traces. Coloured boxes mark behaviour mode classification along the flight path (cyan blue = thermal soaring, pink = display). Behaviour modes were assigned using a 5-second rolling window over altitude change and standard deviation, combined with a heading-rate threshold of 45°/5s to distinguish circling (thermal soaring) from directional (glide/lift) movement. Behavioural thresholds were defined by visual comparison against raw altitude and heading traces across multiple example flights.
